# Development and internal validation of a diagnostic prediction model for osteoporosis in elderly people living with HIV receiving antiretroviral therapy: a cross-sectional study

**DOI:** 10.1186/s12879-026-13670-3

**Published:** 2026-06-26

**Authors:** Chuan Qin, Jinhua Gu, Peng Zhang, Tao Chen, Shenglin Mo, XinMei Meng, Wenming He, Fu Kai, Jiaguang Hu, Zhongsheng Jiang

**Affiliations:** 1 Department of Infectious Diseases, Liuzhou People’s Hospital Affiliated to Guangxi Medical University, Liuzhou, 545006 China; 2 Emergency Department, Liuzhou Traditional Chinese Medicine Hospital, Liuzhou, 545001 China

**Keywords:** Osteoporosis, People living with HIV (PLWH), Nomogram, Calibration curve, Decision curve

## Abstract

**Background:**

Prolonged antiretroviral therapy (ART) in people living with HIV (PLWH) is associated with progressive bone mineral density (BMD) loss and an elevated risk of osteoporosis and fragility fractures. However, validated diagnostic tools for osteoporosis tailored to elderly PLWH remain scarce. This study aimed to develop and internally validate a nomogram-based diagnostic prediction model for osteoporosis in elderly PLWH (postmenopausal women and men aged ≥ 50 years) receiving ART.

**Methods:**

This retrospective cross-sectional study enrolled elderly PLWH who had initiated ART at a single center between September 2010 and July 2022 and had received ART for ≥ 12 months. BMD was assessed by dual-energy X-ray absorptiometry (DXA) during a cross-sectional survey conducted from January to June 2022, and osteoporosis was defined according to the World Health Organization criteria (T-score ≤ − 2.5). Predictor selection was performed using the least absolute shrinkage and selection operator (LASSO) regression. Multivariable logistic regression was subsequently applied to construct a diagnostic prediction nomogram. Model performance was evaluated using the concordance index (C-index), calibration plots, and decision curve analysis (DCA). Internal validation was conducted using bootstrapping with 1,000 resamples.

**Results:**

Among 256 eligible patients, 102 (39.8%) were diagnosed with osteoporosis. LASSO regression identified six predictors with nonzero coefficients, and one additional predictor (baseline CD4 count) was incorporated based on clinical evidence, yielding a seven-variable nomogram: Sex, Ratio of Age and BMI, Duration of ART, Baseline CD4 + count, tenofovir disoproxil fumarate (TDF) regimen, β-C-terminal telopeptide of type I collagen (β-CTX), and procollagen type I N-terminal propeptide (PINP). The model achieved a C-index of 0.709 (95% confidence interval [CI]: 0.645–0.773) with good calibration (mean absolute error: 0.014). Internal validation yielded a bias-corrected C-index of 0.695. DCA demonstrated positive net clinical benefit across threshold probabilities ranging from 0.0 to 0.7.

**Conclusion:**

We developed and internally validated a nomogram incorporating seven clinical and biochemical predictors including Ratio of Age and BMI, Duration of ART, TDF Regimen, β-CTX, PINP, Sex and Baseline CD4 count for the diagnosis of osteoporosis in elderly PLWH receiving ART. The model demonstrated acceptable discrimination, satisfactory calibration and favorable clinical utility. External validation in independent, multi-center cohorts is warranted before clinical implementation.

**Supplementary Information:**

The online version contains supplementary material available at https://doi.org/10.1186/s12879-026-13670-3.

## Introduction

Human immunodeficiency virus (HIV) infection remains a major global public health challenge. As of 2024, approximately 40.8 million individuals are living with HIV worldwide, including an estimated 1.355 million in China [1]. The widespread implementation of antiretroviral therapy (ART) has substantially reduced HIV-related morbidity and mortality and extended life expectancy among people living with HIV (PLWH) [2]. However, this increased longevity has been accompanied by a rising burden of non-AIDS-defining conditions, including metabolic bone disease.

Accumulating evidence indicates that PLWH have a significantly elevated prevalence of osteoporosis and fragility fractures compared with the general population. The pathogenesis of bone loss in this population is multifactorial, involving both modifiable and non-modifiable risk factors. These include advanced age, low body mass index (BMI), low CD4 count, diabetes mellitus, inadequate calcium and vitamin D intake, physical inactivity, smoking, direct viral effects on bone cells, and adverse skeletal effects of certain antiretroviral agents-particularly tenofovir disoproxil fumarate (TDF), and so on [3–5].

Despite the well-recognized burden of osteoporosis in PLWH, validated risk prediction tools tailored to this population remain limited, particularly for elderly patients who bear the highest fracture risk. Existing general-population fracture risk assessment instruments, such as FRAX, do not account for HIV-specific risk factors and may underestimate fracture risk in this group [6]. Therefore, the development of an HIV-specific osteoporosis risk prediction model that integrates clinical, virological, and metabolic parameters is of considerable clinical importance.

The objective of this study was to develop and internally validate a nomogram for diagnostic predicting osteoporosis risk in elderly PLWH receiving ART, incorporating demographic characteristics, treatment-related factors, bone turnover markers and metabolic indicators.

## Methods

### Study design and ethical approval

This cross-sectional study was conducted at the ART Clinic of the Department of Infectious Diseases, Liuzhou People’s Hospital Affiliated to Guangxi Medical University, Guangxi, China. The study protocol was approved by the Medical Ethics Committee of Liuzhou People’s Hospital (Ethics Approval Number: KY2022-059-01). All participants provided informed consent. Patients received treatment adherence education and counseling on smoking cessation, alcohol avoidance and dietary health.

### Study population

Patients who initiated ART between September 2010 and July 2022 were screened for eligibility. The inclusion criteria were as follows: (1) confirmed diagnosis of HIV/AIDS according to the Chinese Guidelines for HIV/AIDS Diagnosis and Treatment [7–11]; (2) postmenopausal women or men aged ≥ 50 years; (3) ART duration ≥ 12 months; and The exclusion criteria were: (1) treatment adherence < 90%; (2) concomitant conditions known to affect BMD, such as multiple myeloma, bone metastases, or thyroid disorders; (3) prior use of bone-protective medications, including calcium supplements, vitamin D, or bisphosphonates, at any point before the cross-sectional survey.

**Fig. 1:**
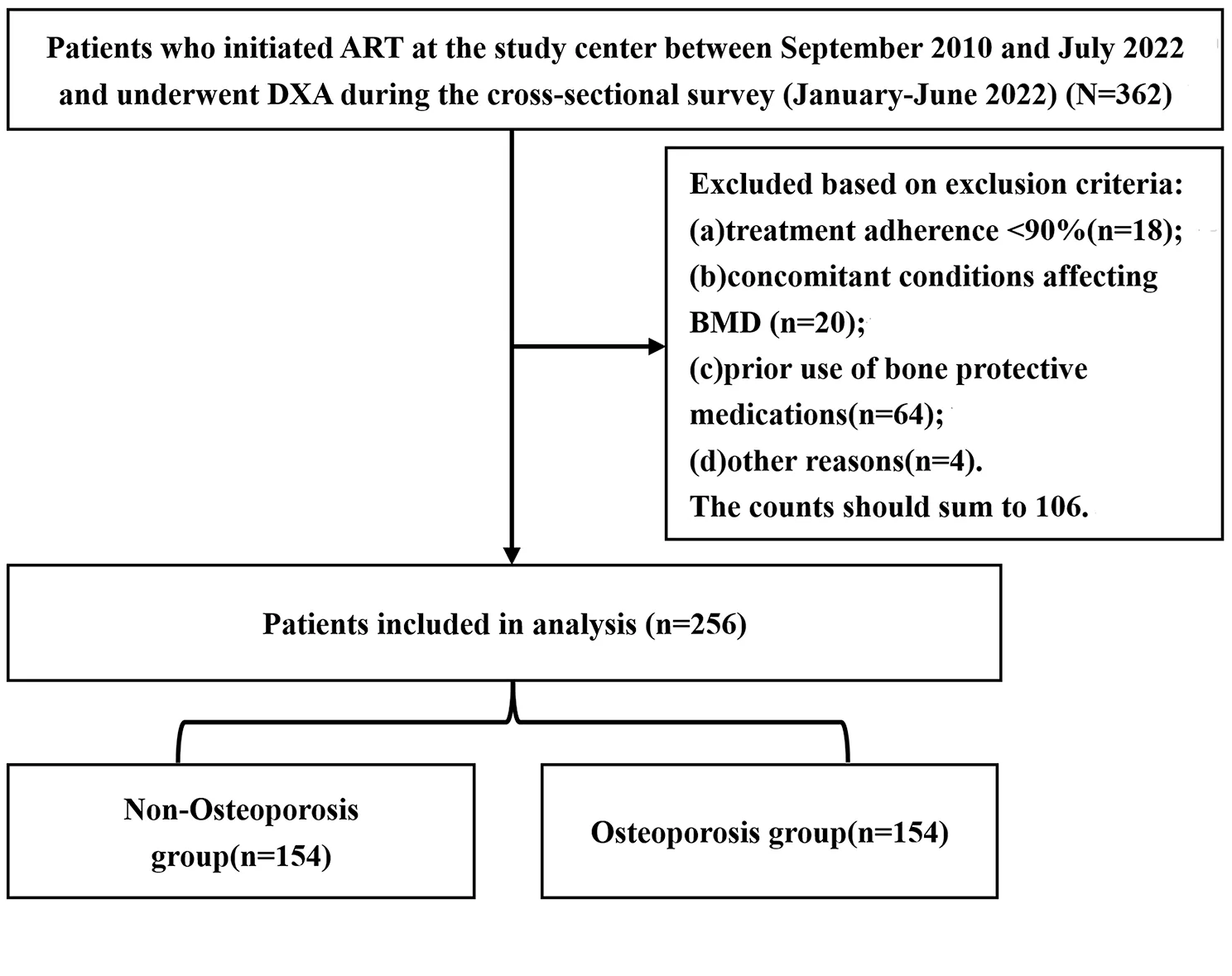
Flowchart of patient screening, enrollment, and group allocation. Between September 2010 and July 2022, 362 elderly PLWH (postmenopausal women and men aged ≥ 50 years) who had initiated ART at the study center were screened during a cross-sectional survey conducted from January to June 2022. After applying the exclusion criteria, 256 patients were included in the final analysis. Osteoporosis was defined as a lumbar spine BMD T-score ≤ − 2.5 measured by DXA. ART, antiretroviral therapy; BMD, bone mineral density; DXA, dual-energy X-ray absorptiometry; PLWH, people living with HIV

Osteoporosis was defined according to the World Health Organization criteria as a BMD T-score ≤ − 2.5 at the lumbar spine, measured by dual-energy X-ray absorptiometry (DXA) [6, 12]. Osteopenia was defined as a T-score between − 1.0 and − 2.5 [13, 14](Figure 1).

### Data collection

All 256 patients included in this study were ART-naïve at the time of treatment initiation (i.e., had no prior exposure to antiretroviral agents before their first ART prescription). Variables were classified into two categories based on the time point of measurement:

**Historical/baseline variables**, recorded at ART initiation and extracted retrospectively from medical records, included: sex, age, height, weight, BMI, baseline ART regimen, baseline CD4 count (cells/µL), and baseline HIV RNA (copies/mL).

**Concurrent variables**, measured during a cross-sectional survey conducted from January 2022 to June 2022, included: lumbar spine BMD T-score assessed by DXA, serum low-density lipoprotein cholesterol (LDL-C), and bone turnover markers- procollagen type I N-terminal propeptide (PINP), β-C-terminal telopeptide of type I collagen (β-CTX), and 25-hydroxyvitamin D2 (25(OH)D2).

### Sample size estimation

The minimum sample size was estimated using the formula n = (10 × k) / p, where k represents the number of candidate predictor variables and p represents the incidence of the target event [15, 16]. With up to 10 candidate predictors and an estimated osteoporosis prevalence of 40% based on preliminary data, the minimum required sample size was 250 patients.

### Statistical analysis

All statistical analyses were performed using R version 4.4.2 (R Foundation for Statistical Computing, Vienna, Austria). Continuous variables of normal distribution are expressed as mean ± standard deviation, while continuous variables with non-normal distributions were expressed as medians with interquartile ranges [M (Q1, Q3)]. Categorical variables were presented as frequencies and percentages. Missing data (baseline HIV RNA and baseline CD4 count were missing for 11 patients [4.3%]) were imputed using multiple imputation.

Predictor selection was performed using LASSO regression with fivefold cross-validation. The optimal regularization parameter was selected based on the minimum deviance criterion (λ min). Variables with nonzero coefficients at λ min were retained as candidate predictors. Consistent with established prediction modeling methodology, variables selected by LASSO were retained in subsequent analyses regardless of individual P-value significance in unpenalized logistic regression, as individual P-values should not be used to further eliminate LASSO-selected variables [17]. Collinearity was assessed using the variance inflation factor (VIF); all independent variables had VIF values below 5, indicating no substantial multicollinearity.

LASSO identified six predictors with nonzero coefficients: Ratio of Age and BMI, Duration of ART, TDF Regimen, β-CTX, PINP and Sex. Ln (Baseline CD4 count) was incorporated into the model based on a priori clinical knowledge, given the well-established association between low Baseline CD4 count and accelerated BMD loss following ART initiation [18, 19].

A multivariable logistic regression model was constructed using the seven final predictors. Results were expressed as odds ratios (ORs) with 95% confidence intervals (CIs). A nomogram was generated to visualize the diagnostic prediction model. Model discrimination was assessed using the C-index and the area under the receiver operating characteristic (ROC) curve. Calibration was evaluated using calibration plots comparing predicted probabilities against observed outcomes. Clinical utility was assessed by decision curve analysis (DCA), which quantifies the net benefit at various threshold probabilities by weighing the relative harms of false-positive and false-negative classifications. Internal validation was performed using bootstrapping with 1,000 resamples to estimate a bias-corrected C-index [17]. A two-sided P-value < 0.05 was considered statistically significant.

## Results

### Patient characteristics

A total of 256 elderly PLWH met the inclusion criteria, comprising 143 males (55.9%) and 113 females (44.1%), with a mean age of 60.53 ± 9.02 years. Among these, 102 patients (39.8%) had osteoporosis and 154 (60.2%) did not. The median lumbar spine T-score was (− 2.20) {interquartile range [IQR]: [ (− 2.88) to (− 1.03)]} overall, (− 1.40) [[IQR: (− 2.10) to (− 0.30)] in the non-osteoporosis group, and(− 3.00)[ (IQR: (− 5.60) to (− 2.50)) in the osteoporosis group. All patients had achieved virological suppression (HIV RNA < 200 copies/mL) at the time of assessment. In this study, data on baseline HIVRNA and baseline CD4 count were missing for 11 patients (4.3%), and multiple imputation was used to impute all missing values. The demographic and clinical characteristics of both groups are presented in Tables 1 and 2.

**Table 1:** Baseline categorical characteristics of the study population stratified by osteoporosis status (*N* = 256). Data are presented as n (%). Osteoporosis was defined as a lumbar spine BMD T-score ≤ − 2.5 measured by DXA. Group comparisons were performed using the chi-square test

Demographic characteristics	*N* (%)			
	non-osteoporosis group (*n* = 154)	osteoporosis group (*n* = 102)	X^2^	*P*
Sex			1.637	0.201
female	63(40.91%)	50(49.02%)		
male	91(59.09%)	52(50.98%)		
TDF Regimen			6.406	0.011
No(including non-TDF)	100(64.94%)	50(49.02%)		
Yes(including TDF))	54(35.06%)	52(50.98%)		

Abbreviations: ART, antiretroviral therapy; TDF, tenofovir disoproxil fumarate

**Table 2:** Clinical characteristics of the study population stratified by osteoporosis status (*N* = 256)[Non-osteoporosis group (*n* = 154) and Osteoporosis group (*n* = 102)]. Osteoporosis was defined as a lumbar spine bone mineral density T-score ≤ − 2.5 measured by dual-energy X-ray absorptiometry

Clinical characteristics	non-osteoporosis group($\bar x \pm s$)	osteoporosis group($\bar x \pm s$)	t	*P*
Ratio of Age and BMI	2.689 ± 0.549	2.816 ± 0.526	1.851	0.065
Duration of ART (months)	87.490 ± 51.460	95.900 ± 52.880	1.266	0.207
Log(Baseline HIV RNA)	4.609 ± 0.938	4.559 ± 0.969	0.412	0.681
25(OH)D2(nmol/L)	2.540 ± 0.978	2.602 ± 1.048	0.481	0.631
β-CTX(pg/ml)	0.411 ± 0.222	0.566 ± 0.320	4.589	< 0.001
P1NP(ng/ml)	62.422 ± 30.581	78.608 ± 34.660	3.831	< 0.001
LDL-C(mmol/L)	3.117 ± 0.975	3.213 ± 0.962	0.772	0.444
Ln(Baseline CD4 count)	4.751 ± 1.176	4.589 ± 1.246	1.052	0.294

Abbreviations: BMI, body mass index; ART, antiretroviral therapy; HIV RNA, human immunodeficiency virus ribonucleic acid; CD4, CD4-positive T-lymphocyte count;25(OH)D2, 25-hydroxyvitamin D2; β-CTX, β-C-terminal telopeptide of type I collagen; PINP, procollagen type I N-terminal propeptide; LDL-C, low-density lipoprotein cholesterol

Because this study spanned up to 12 years, during which antiretroviral therapy regimens underwent significant changes - from predominantly non-nucleoside reverse transcriptase inhibitors (NNRTIs) in the early phase (between 2010 and 2014) to increasing use of protease inhibitors (PIs) (between 2015 and 2017) and integrase strand transfer inhibitors (INSTIs) (between 2018 and 2022) - temporal heterogeneity may have been introduced. The incidences of osteoporosis in three different stages of antiretroviral therapy, represented by NNRTIs, PIs, and INSTIs, were 40.82% (40/98), 46.88% (30/64), and 34.04% (32/94), respectively. A stratified analysis across three ART eras showed no significant difference in osteoporosis prevalence (χ² = 2.769, *P* = 0.262), but this does not fully exclude era-specific confounding.

### Feature selection

**Fig. 2:**
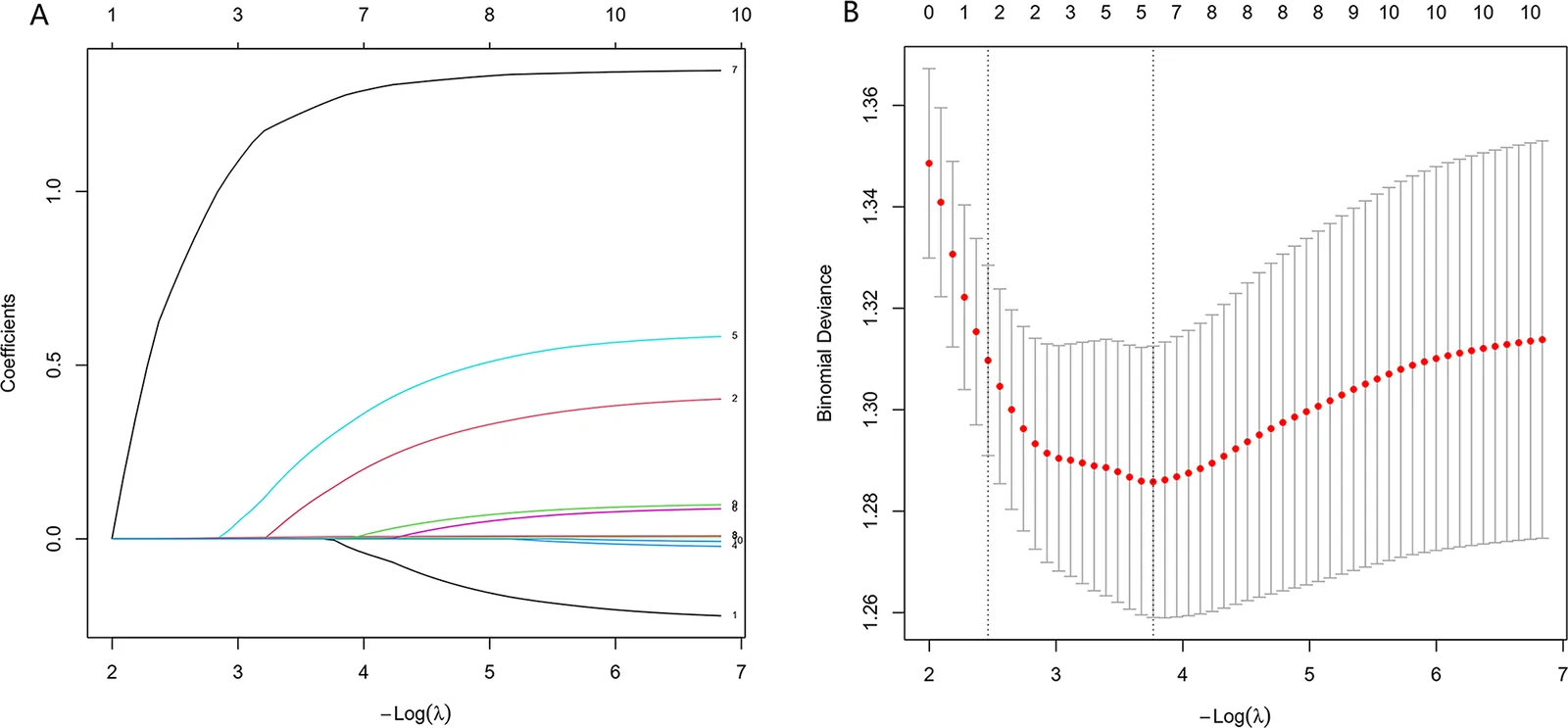
Predictor selection using LASSO logistic regression. (**A**) LASSO coefficient profiles for 10 candidate predictors. Each line represents the trajectory of a single variable’s coefficient as a function of –Log(λ). (**B**) Selection of the optimal tuning parameter (λ) via fivefold cross-validation. Binomial deviance is plotted against –Log(λ). The left vertical dashed line indicates λ_1se; the right vertical dashed line indicates λ_min = 0.02315, at which six predictors retained nonzero coefficients. LASSO, least absolute shrinkage and selection operator

Of 10 candidate demographic, clinical, and laboratory variables, LASSO regression identified six predictors with nonzero coefficients at λ min = 0.02315 (Fig. 2A and B): Ratio of Age and BMI, Duration of ART, TDF Regimen, β-CTX, PINP, and Sex. As described in the Methods section, Ln (Baseline CD4 count) was incorporated based on clinical evidence.

Multivariable logistic regression analysis of the seven final predictors yielded the following results. β-CTX (OR = 4.22; 95% CI: 1.15–15.53; *P* = 0.030) and Duration of ART (OR = 1.01; 95%CI: 1.00–1.01; *P* = 0.045) were statistically significantly associated with osteoporosis. TDF Regimen showed a trend toward association with higher odds of osteoporosis (OR = 1.76; 95% CI: 0.96–3.21; *P* = 0.066). Ratio of Age and BMI (OR = 1.50; 95%CI: 0.90–2.50; *P* = 0.120), PINP (OR = 1.01; 95% CI: 1.00–1.02; *P* = 0.116), Sex (OR = 0.80; 95% CI: 0.45–1.42; *P* = 0.455), and Ln(Baseline CD4 count) (OR = 0.98; 95% CI: 0.77–1.24; *P* = 0.842) did not reach conventional statistical significance but were retained in the model based on the LASSO selection framework and clinical relevance. The VIF values for all predictors ranged from 1.105 to 1.578, indicating no substantial multicollinearity (Table 3).

**Table 3:** Multivariable logistic regression analysis of predictors for osteoporosis in elderly PLWH receiving ART. Candidate predictors were identified using LASSO regression with fivefold cross-validation (λ min = 0.02315). Variables with nonzero coefficients were entered into multivariable logistic regression; Ln (baseline CD4 count) was additionally incorporated based on clinical evidence (see Methods)

Intercept and variable	Prediction model			
	β	Odds ratio (95% CI)	*P*-value	VIF
Intercept	-3.3255	0.0359(0.0004–0.3221)	0.0030	-
Ratio of Age and BMI	0.4056	1.5000(0.8997–2.5013)	0.1200	1.105
Duration of ART	0.0057	1.0058(1.0001–1.0114)	0.0453	1.240
TDF Regimen	0.5645	1.7586(0.9626–3.2123)	0.0664	1.277
β-CTX	1.4405	4.2228(1.1485–15.5263)	0.0301	1.578
PINP	0.0084	1.0084(0.9979–1.0190)	0.1155	1.536
Sex	-0.2199	0.8043(0.4544–1.4237)	0.4549	1.268
Ln(Baseline CD4 count)	-0.0240	0.9763(0.7712–1.2358)	0.8417	1.204

Abbreviations: β, regression coefficient; CI, confidence interval; BMI, body mass index; ART, antiretroviral therapy; HIV RNA, human immunodeficiency virus ribonucleic acid; TDF, tenofovir disoproxil fumarate; β-CTX, β-C-terminal telopeptide of type I collagen; PINP, procollagen type I N-terminal propeptide; LDL-C, low-density lipoprotein cholesterol; LASSO, least absolute shrinkage and selection operator, VIF, variance inflation factor. VIF is not applicable to the intercept

### Development of the nomogram

**Fig. 3:**
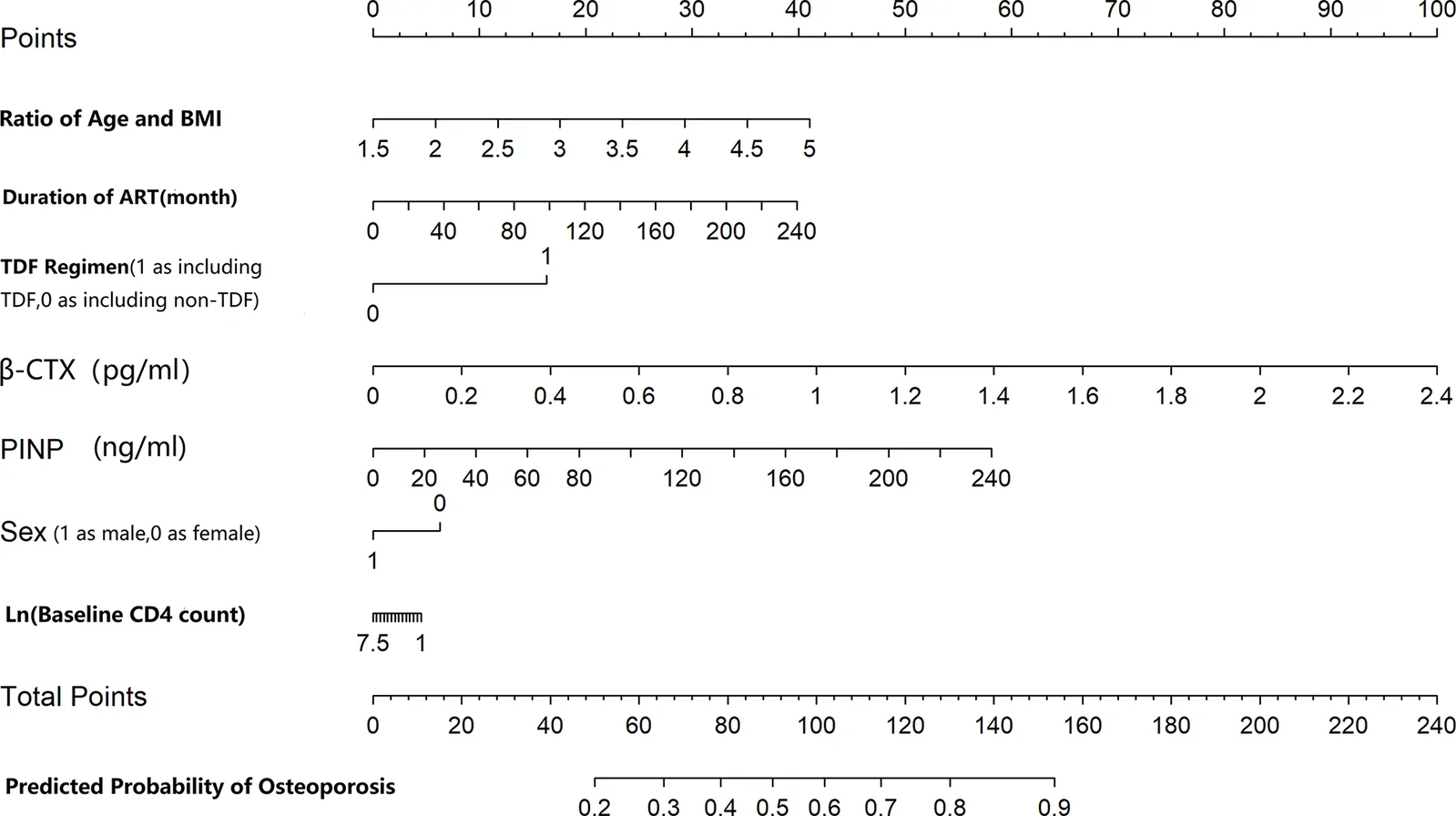
Diagnostic nomogram for predicting osteoporosis in elderly PLWH receiving ART. To estimate the probability of osteoporosis, locate the value for each predictor on its respective axis, draw a vertical line upward to the “Points” axis to obtain the corresponding score, and sum the scores for all seven predictors. The total points value is mapped to the bottom axis to determine the predicted probability. Higher total scores indicate greater predicted probability. Binary coding: Sex (0 = female, 1 = male); TDF Regimen (0 = including non-TDF, 1 = including TDF). BMI, body mass index; ART, antiretroviral therapy; TDF, tenofovir disoproxil fumarate; β-CTX, β-C-terminal telopeptide of type I collagen (pg/mL); PINP, procollagen type I N-terminal propeptide (ng/mL)

A nomogram incorporating the seven identified predictors was constructed to estimate individual osteoporosis risk (Fig. 3). Each predictor is assigned a point value on the nomogram, and the sum of these values corresponds to a predicted probability of osteoporosis.

### Model performance

**Fig. 4:**
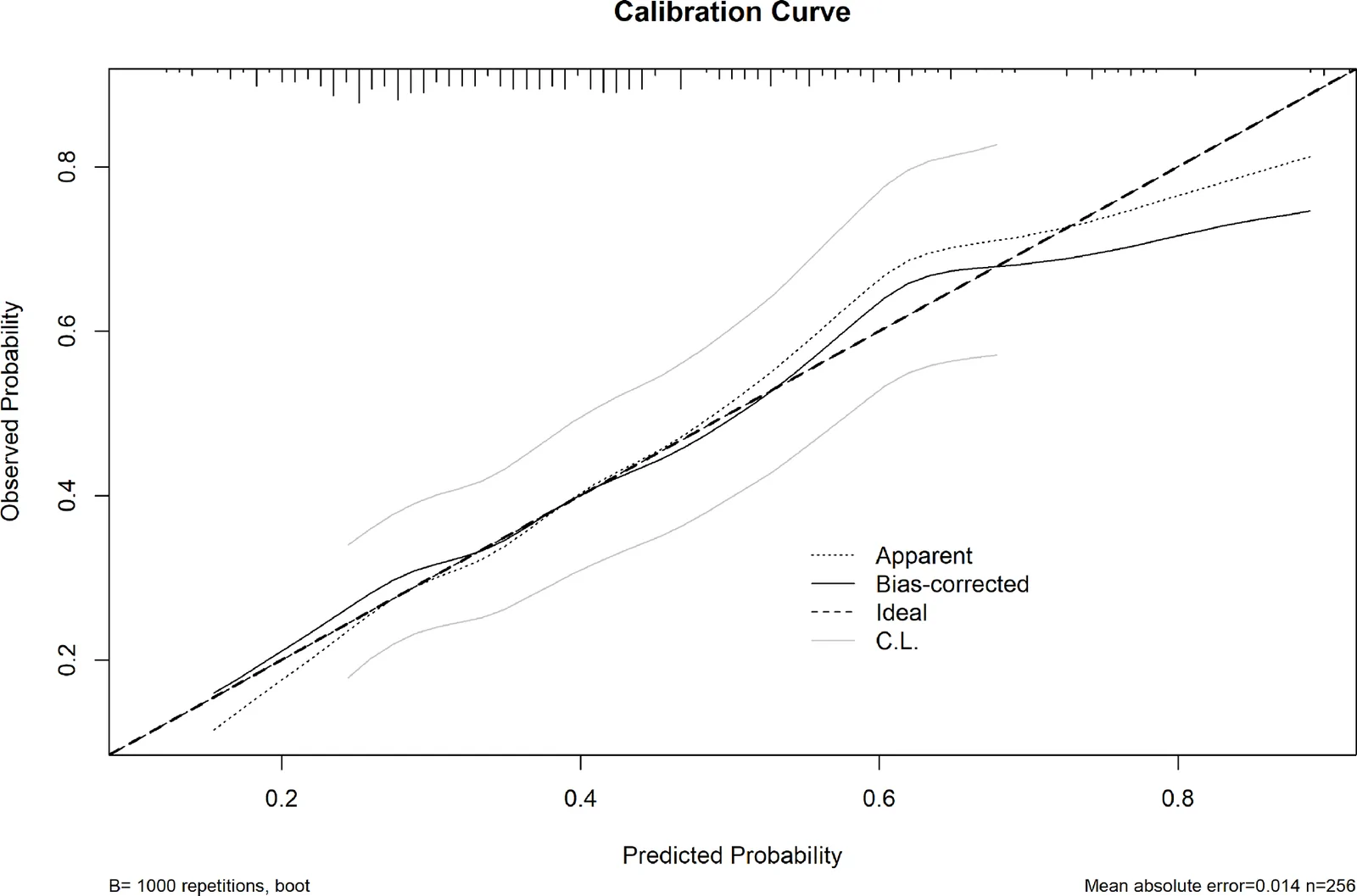
Calibration curve for the osteoporosis risk prediction nomogram. The x-axis represents the nomogram-predicted probability of osteoporosis, and the y-axis represents the observed probability of osteoporosis. The diagonal dashed line represents perfect calibration (i.e., predicted probability equals observed probability). The solid line depicts the actual performance of the nomogram; closer alignment with the diagonal indicates better calibration. The apparent calibration curve (without correction) and the bias-corrected curve (adjusted via bootstrapping with 1,000 resamples) are shown. The mean absolute error between predicted and observed probabilities was 0.014(*n* = 256)

The C-index for the nomogram was 0.709 (95% CI: 0.645–0.773; *P* < 0.001), indicating acceptable discriminative ability. The calibration curve demonstrated good agreement between predicted probabilities and observed outcomes, with a mean absolute error (MAE) of 0.014, indicating excellent calibration (Fig. 4). Bootstrap internal validation yielded a bias-corrected C-index of 0.695, suggesting adequate model stability. The area under the curve (AUC) of the nomogram was 0.709 (95% CI: 0.645–0.773; *P* < 0.001). The corresponding receiver operating characteristic curve is presented in Additional file 1: Figure S1.

### Clinical utility

**Fig. 5:**
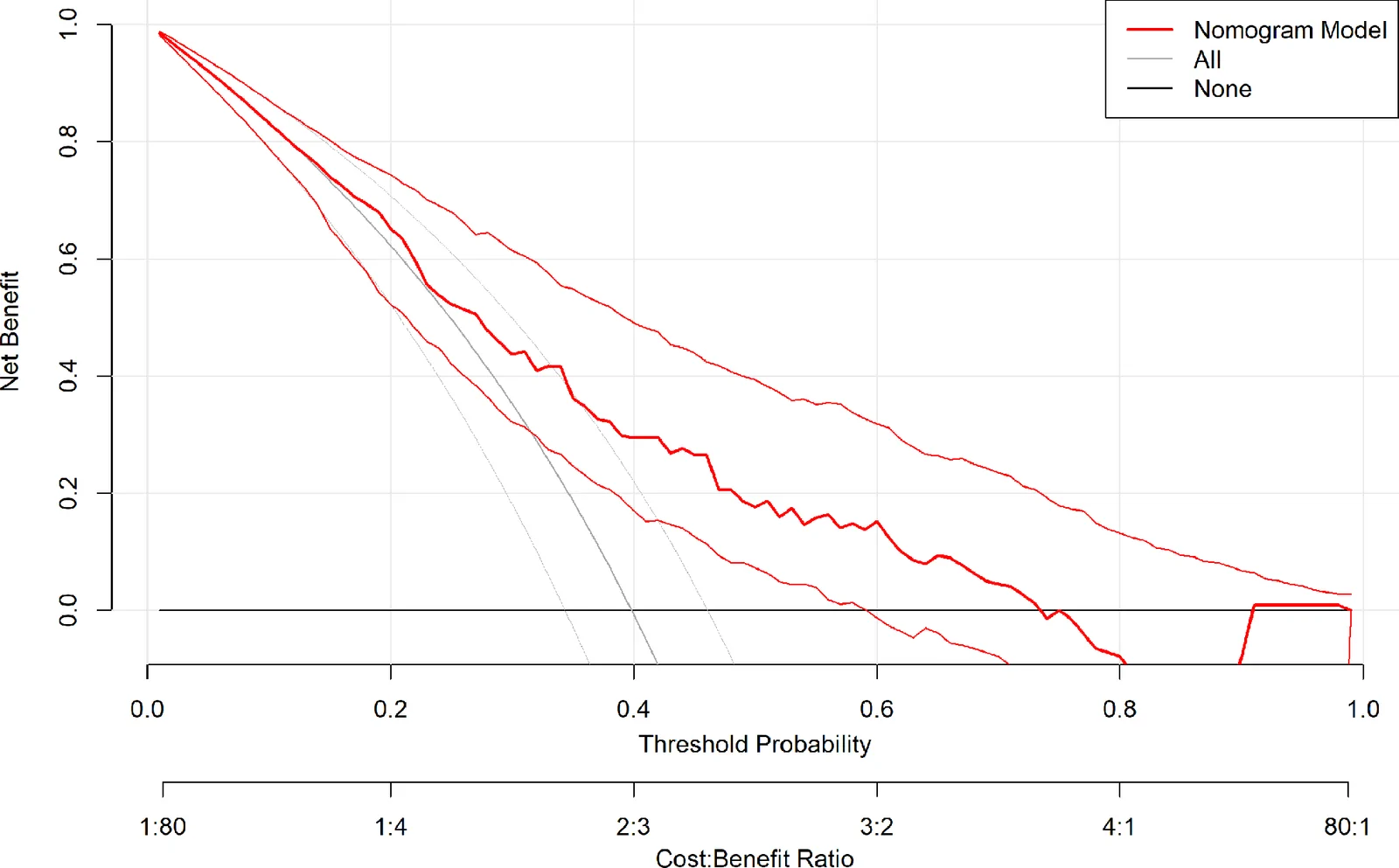
Decision curve analysis for the osteoporosis risk prediction nomogram. Decision curve analysis for the diagnostic nomogram. The x-axis represents the threshold probability for initiating intervention, and the y-axis represents net benefit. The red line represents the nomogram model [the thinner red line represents a confidence band]. The gray line represents the “treat-all” strategy, and the black horizontal line represents the “treat-none” strategy. The nomogram demonstrated positive net benefit at threshold probabilities from approximately 0.0 to 0.7. The secondary x-axis displays the corresponding cost-to-benefit ratio

Decision curve analysis (DCA) revealed that the nomogram yielded positive net clinical benefit across a broad threshold probability range (0.0–0.7). Specifically, the model outperformed both the “treat-all” and “treat-none” strategies when the threshold probability was below 0.7, with the maximum net benefit observed between 0.0 and 0.5. This indicated that applying the nomogram to guide clinical decisions within this range could optimize patient stratification and reduce unnecessary interventions. **(**Fig. 5**)**

## Discussion

### Principal findings

In this retrospective cross-sectional study of 256 elderly PLWH receiving ART, we developed and internally validated a diagnostic nomogram integrating seven predictors: Ratio of Age and BMI, Duration of ART, TDF Regimen, β-CTX, PINP, Sex, and Ln (Baseline CD4 count). The model demonstrated acceptable discrimination (C-index: 0.709; bias-corrected: 0.695) and excellent calibration (MAE: 0.014). Decision curve analysis showed that the nomogram achieved higher net benefit than the treat-all and treat-none strategies across threshold probabilities from 0.0 to 0.7, supporting its potential clinical utility for identifying elderly PLWH at high risk of osteoporosis.

### Demographic factors

The Ratio of Age and BMI emerged as an independent predictor of diagnosis osteoporosis (OR = 1.50; 95% CI0.90-2.50; *P* = 0.12), integrating the dual effects of advancing age and low nutritional status. Both advanced age and low BMI are well-established risk factors for osteoporosis in the general population and in PLWH specifically [14]. A cross-sectional study from Zimbabwe involving 393 women aged 40–60 years demonstrated that advanced age, low body weight, and HIV infection were the strongest predictors of reduced femoral neck BMD [20]. Similarly, a Thai study of HIV-infected men over 60 years of age identified low BMI as an independent predictor of low BMD at both the lumbar spine and femoral neck [21].

The biological plausibility of this composite indicator is supported by evidence of fat-bone metabolic crosstalk. Evidence suggests that hormonal interactions between adipose tissue and bone mass following ART initiation may be particularly detrimental in individuals with low adiposity, potentially impairing leptin-mediated support of bone formation [22]. The Ratio of Age and BMI may therefore serve as a clinically accessible marker for identifying individuals with a higher probability of osteoporosis.

In the multivariable analysis, male sex showed a non-significant trend toward a protective association (OR = 0.80; 95% CI: 0.45–1.42; *P* = 0.455). Although this finding did not reach statistical significance, the direction of the association is consistent with published literature. A study using quantitative computed tomography found that bone structural damage was more extensive and severe in women living with HIV than in men [23]. Several mechanisms may underlie this observation. First, postmenopausal estrogen deficiency leads to substantially higher rates of bone loss in women compared with age-matched men, who do not experience an equivalent hormonal withdrawal. Notably, even among men, estradiol rather than testosterone has been identified as the key independent predictor of bone mineral density in HIV-infected individuals aged ≥ 50 years [24], suggesting that estrogen-mediated pathways are central to bone homeostasis in both sexes. Data from the Women’s Interagency HIV Study cohort demonstrated that HIV infection and menopausal status have independent and additive negative effects on bone mineral density [25]. These findings suggest that the observed trend reflects the combined influence of postmenopausal bone loss, sex hormone levels, and sex-specific differences in bone architecture [26].

### Factors related to antiviral treatment

Use of a TDF-containing regimen was associated with 1.76-fold higher odds of osteoporosis (OR = 1.76; 95% CI: 0.96–3.21; *P* = 0.066), although this association did not reach conventional statistical significance. The proposed mechanism centers on proximal renal tubular dysfunction: TDF accumulates intracellularly via OAT1/OAT3 transporters, induces mitochondrial toxicity, and downregulates the NaPi-IIa transporter, leading to urinary phosphate wasting. This may trigger compensatory reductions in 1,25-(OH)₂D synthesis, decreased intestinal calcium absorption, secondary hyperparathyroidism, and osteoclast activation [27]. Importantly, this renal-bone toxicity appears at least partially reversible; a multi-center randomized trial in China demonstrated significant improvement in hip BMD within 48 weeks of switching from TDF-based to bictegravir/emtricitabine/tenofovir alafenamide (B/F/TAF)-based regimens [28].

The results of this study indicated that the Duration of ART in PLWH was associated with 1.01-fold higher odds of diagnosis osteoporosis in PLWH (OR = 1.01;95%CI1.00-1.01;*P* = 0.045). A Japanese observational study reported a significantly increased cumulative probability of osteoporotic fractures after ≥ 5 years of TDF exposure [29]. Similarly, a Thai prospective study demonstrated an independent association between TDF use and ≥ 5% BMD decline over 5 years [30]. The underlying mechanism may involve the senescence-associated secretory phenotype (SASP), whereby chronically activated immune cells secrete pro-inflammatory and pro-osteoclastogenic factors (e.g., IL-6, TNF-α, RANKL), creating a persistent microenvironment that favors bone resorption [31].

Baseline CD4 count showed a non-significant trend toward a protective association (OR = 0.98; 95% CI: 0.77–1.24; *P* = 0.842). Although this variable was not retained by LASSO, it was included based on well-established clinical evidence. The association between low baseline CD4 levels and accelerated bone loss may be related to immune reconstitution-associated bone loss (IRBL) following ART initiation. Patients with lower baseline CD4 levels experience a greater magnitude of immune reconstitution, accompanied by activation of osteoclastogenic cytokines including RANKL and TNF-α, leading to more pronounced inflammatory bone resorption [19]. Furthermore, HIV-induced dysfunction of B cells and T cells leads to an imbalance in the OPG/RANKL axis, thereby promoting osteoclastogenesis [32]. Similar associations have been reported in both domestic and international studies [18–34].

### Bone turnover markers (β-CTX and P1NP)

β-CTX was the strongest predictor in the model (OR = 4.22; 95% CI: 1.15–15.53; *P* = 0.030), while PINP showed a trend toward association (OR = 1.01; 95% CI: 1.00–1.02; *P* = 0.116). These findings are consistent with international guidelines recommending β-CTX and PINP as the preferred indicators of bone resorption and formation, respectively [35, 36]. Elevated β-CTX reflects accelerated osteoclast-mediated bone resorption, while elevated PINP may paradoxically indicate compensatory osteoblast activation in the context of high bone turnover.

Longitudinal studies support the predictive value of these markers. Longitudinal evidence has shown that women with persistently high bone turnover experienced significantly greater BMD loss over 5 years [37]. In the HIV-specific context, the SECOND-LINE study demonstrated that early PINP elevation following ART switch was a predictor of subsequent hip bone loss [38].

### Comparison with existing models

The discriminative performance of our model (C-index: 0.709) favorably with a machine learning-based model (AUC: 0.615) that lacked bone turnover markers [39]. Another model (AUC: 0.835) achieved higher discrimination but was restricted to male patients [40].

An XGBoost-based fracture risk assessment tool was developed using clinical data from 1,045 PLWH, achieving AUC values of 0.984 (training) and 0.979 (external validation) [33]. However, their model requires lumbar spine BMD as an input variable, which presupposes DXA availability and thus limits its utility as a pre-screening tool. In contrast, our nomogram is designed to identify individuals who should be prioritized for DXA referral, addressing a complementary clinical need. Our model’s inclusion of biochemical markers reflecting bone metabolic status provides a more mechanistically informed risk assessment, although its application is contingent on the availability of bone turnover marker testing.

### Strengths and limitations

The principal strengths of this study include the integration of multiple pathophysiological dimensions - demographic, treatment-related, immunological, and metabolic-into a single diagnostic prediction model, the use of LASSO regression to mitigate overfitting, and the inclusion of bone turnover markers, which have been underrepresented in prior HIV-related osteoporosis models.

Several limitations must be acknowledged. First, the cross-sectional design precludes the establishment of temporal or causal relationships between predictors and the outcome; all observed associations should be interpreted accordingly, and prospective validation is essential. Second, external validation in an independent cohort was not performed. Although bootstrap internal validation demonstrated adequate model stability (bias-corrected C-index: 0.695), generalizability to other populations remains uncertain. Our research group is currently establishing multi-center collaborations for external validation. Third, a direct comparison with established fracture risk tools such as FRAX was not conducted, as the required parameters were not systematically recorded in patients’ medical records. Fourth, the sample size of 256 patients with 102 events provides an events-per-variable (EPV) ratio of 14.6, which exceeds the minimum threshold of 10 recommended in the prediction modeling literature [15] but falls below the more conservative recommendation of 20 [16]. The use of LASSO regularization partially mitigates overfitting risk, though coefficient estimates may exhibit instability. Fifth, Ln (Baseline CD4 count) was incorporated into the model based on clinical judgment rather than LASSO selection. Finally, the exclusion of patients who had used bone-protective agents may have underestimated the true prevalence of osteoporosis in the broader PLWH population.

### Clinical implications

Based on these findings, clinicians managing elderly PLWH on long-term ART should consider the following: (1) for patients with traditional risk factors (female sex, advanced age, low BMI), ART regimens with favorable bone safety profiles (e.g., tenofovir alafenamide [TAF]- or dolutegravir [DTG]-based regimens) may be preferred; (2) routine monitoring of bone turnover markers (β-CTX, PINP) may facilitate the identification of individuals with accelerated bone metabolism who warrant DXA referral.

Regarding threshold selection for clinical decision-making, the DCA results suggest that the nomogram provides net clinical benefit when applied at threshold probabilities between 0.0 and 0.7. Within this range, clinicians may select lower thresholds (e.g., 0.2–0.3) in settings that prioritize sensitivity and early identification, or moderate thresholds (e.g., 0.4–0.5) in settings that prioritize specificity and resource conservation. Thresholds exceeding 0.7 are not recommended, as the model’s net benefit approaches that of the treat-none strategy at these levels. These thresholds are empirically derived from single-center data and require validation in diverse clinical settings before adoption.

## Conclusion

This study developed and internally validated a seven-variable diagnostic nomogram for osteoporosis in elderly PLWH receiving ART. The model, incorporating demographic, treatment-related, immunological, and metabolic predictors, demonstrated acceptable discrimination (C-index: 0.709), excellent calibration (MAE: 0.014), and favorable clinical utility across a broad range of threshold probabilities. The nomogram may serve as a practical tool for individualized risk stratification and clinical decision-making regarding DXA referral in this population. Prospective external validation in multi-center, independent cohorts is necessary before clinical adoption.

## Supplementary Information

Below is the link to the electronic supplementary material.

Supplementary Material 1

Supplementary Material 2

## Data Availability

The data that support the findings of this study are available from the corresponding author (N.S.) upon reasonable request.

## Abbreviations

HIV: Human Immunodeficiency Virus
AIDS: Acquired Immunodeficiency Syndrome
PLWH: people living with HIV
ART: Antiretroviral Therapy
BMD: Bone Mineral Density
BMI: Body Mass Index
DXA: Dual-Energy X-ray Absorptiometry
WHO: World Health Organization
CD4: Cluster of Differentiation 4
RNA: Ribonucleic Acid
LASSO: least absolute shrinkage and selection operator
LDL-C: Low-density lipoprotein cholesterol
PINP: Procollagen type I N-terminal propeptide
β-CTX: β-C-terminal telopeptide of type I collagen
25(OH)D2: 25-hydroxyvitamin D2
C-index: Concordance index
ROC: Receiver operating characteristic
DCA: Decision curve analysis
TDF: Tenofovir disoproxil fumarate
AUC: Area Under the ROC Curve
SASP: Senescence-associated secretory phenotype
B/F/TAF: Bictegravir/ emtricitabine/ tenofovir alafenamide
NNRTIs: Reverse transcriptase inhibitors
PIs: Protease inhibitors
INSTIs: Integrase strand transfer inhibitors
EFV: Efavirenz
VIF: Variance inflation factor
