# Supplementary material for: Development and internal validation of a diagnostic prediction model for osteoporosis in elderly people living with HIV receiving antiretroviral therapy: a cross-sectional study
Source: BMC Infect Dis. 2026 Jun 26;26:1643. doi: 10.1186/s12879-026-13670-3 (PMC13563893; doi:10.1186/s12879-026-13670-3)
Supplement: Supplementary file 1 [file 12879_2026_13670_MOESM1_ESM.docx]

Additional file 2: Figure S1

Figure S1. Receiver operating characteristic (ROC) curve for the diagnostic nomogram. The area under the curve (AUC) was 0.709 (95% CI: 0.645–0.773). The diagonal dashed line represents chance classification (AUC = 0.5).”
